# Palliative Systemic Therapy in Advanced Thymic Epithelial Tumors in 2026—A Narrative Review

**DOI:** 10.3390/cancers18142354

**Published:** 2026-07-21

**Authors:** Felix C. Saalfeld, Tobias R. Overbeck

**Affiliations:** 1 Department for Internal Medicine I, Medical Faculty and University Hospital Carl Gustav Carus, TUD Dresden University of Technology, Fetscherstr. 74, 01307 Dresden, Saxony, Germany; 2 Thoracic Oncology Group, National Center for Tumor Diseases (NCT) Dresden, Fetscherstr. 74, 01307 Dresden, Saxony, Germany; 3 Department of Hematology and Medical Oncology, University Medical Center Göttingen, Göttingen University, Robert-Koch-Str. 40, 37075 Göttingen, Lower Saxony, Germany; tobias.overbeck@med.uni-goettingen.de; 4 Lung Tumor Center, University Medical Center Göttingen, Göttingen University, Robert-Koch-Str. 40, 37075 Göttingen, Lower Saxony, Germany

**Keywords:** thymoma, thymic carcinoma, systemic therapy, therapy sequence

## Abstract

Palliative systemic therapy for advanced thymic epithelial tumors (TET) is challenging due to scarce evidence and biological heterogeneity. Scientific discussion, as in this review, is limited to cross-trial comparisons of small, non-randomized studies. Platinum-based combination chemotherapy is the standard first-line treatment. Here, we challenge the addition of anthracyclines to platinum in thymoma. We critically appraise new combinations with antiangiogenic drugs and immune checkpoint inhibitors (ICI) in thymic carcinoma and their implications for therapy sequences. We review the data on classic genomics-based targeted therapy that does not benefit most patients and explore established and upcoming biomarkers including KIT and HER2. Finally, we discuss investigational therapies like anti-TROP2 antibody–drug conjugates and proteasome inhibitors, as well as topics and design of future research.

## Simple Summary

Thymic epithelial tumors are ultra-rare diseases and comprise thymoma and thymic carcinoma. Here, we review the current knowledge about drug treatment of such tumors that cannot be removed by surgery or irradiated with the aim of cure. In thymoma, we ask whether anthracyclines should be recommended to all patients given new data about their toxicity to the heart and the extent of their anti-tumor effect. In thymic carcinoma, we discuss how therapies can be used in different sequences, especially when using drugs targeting blood vessels or the immune system (antiangiogenics, immune checkpoint inhibitors). Finally, we identify knowledge gaps and suggest future research aims in areas of drug efficacy, safety, as well as trial conduct and regulation.

## 1. Introduction

Within thoracic oncology, systemic therapy for patients with advanced thymic epithelial tumors (TET) poses unique challenges due to their ultra-low incidence and specific disease biology. Three aspects make it especially difficult to transfer existing evidence directly to clinical scenarios. First, evidence is available only from single-arm phase 2 trials and small retrospective analyses. Comparability of data sets is limited. Heterogeneity between studies is related to different TET entities comprising thymomas (THY) and thymic carcinomas (TC), each with different subtypes, as well as neuroendocrine neoplasms (NEN) [1]. Central pathology review which has been shown to reclassify TET diagnosis in 21–28%of cases [2,3] is not available in several studies. Second, given that complete resection has been associated with improved outcomes even in stage IV TET [4,5,6], the inter-institutional variability in defining primary and induced resectability can be expected to interact with the outcomes of drug trials. Third, advanced TET vary considerably in clinical presentation. As a general rule, TC is more aggressive (5-year overall survival (OS) of inoperable TC: 24% [7]) compared to THY which can be a slow-growing, indolent disease with a better prognosis (5-year OS 58.5% for stage IV [5]). On the other hand, THY is associated with autoimmunity, most notably myasthenia gravis.

In this review, we discuss treatment options for patients with advanced TET, i.e., with TNM stage IV (and selected III) disease not eligible for complete resection (with or without preceding *induction chemotherapy* or curative-intent radiotherapy. According to the International Thymic Malignancy Interest Group (ITMIG) framework [8,9], this involves *exclusive chemotherapy* and *therapy for recurrence.* For the purpose of simplification, we use the common oncologic terminology of first- and second-line palliative systemic therapy. We acknowledge that in case of response, resectability should be re-assessed. Our aim is to critically appraise recently published studies (from 2000 until May 2026 as identified by Google Scholar search using the terms “thymoma” and “thymic carcinoma”) and the current standard-of-care and to identify major issues requiring further investigation. Negative studies with new drugs otherwise not implicated in TET therapy are not reported. Conference abstracts are included and labeled accordingly.

Treatment of thymic NEN is beyond the scope of this review. The therapeutic approach is similar to pulmonary NEN, although differences exist especially regarding disease biology and prognosis [10].

For a definition of standard therapy, we refer to the guidelines of ESMO [11] and NCCN [12]. Due to the relevance of the central review in TET, we indicate whether trials conducted a central pathology (Path+/–) and central radiology review (Rad+/–).

## 2. The Goal of Palliative Systemic Therapy and Associated Trial Endpoints

In contrast to most malignancies, it has not been validly demonstrated that systemic therapy prolongs survival in advanced TET. This is of particular relevance in patients with indolent disease, mostly THY, where the benefits of therapy not always outweigh its toxicity and a watchful waiting strategy may be justified. Symptomatic disease or TC in most cases justify systemic therapy. If symptom control (or induction of resectability) is aimed for in a patient, then the high response rate (ORR) of a drug-in-question might be prioritized over other endpoints. In contrast, in aggressive disease (TC), OS is the most relevant endpoint, whereas in indolent THY with longer duration of treatment, tolerability might be equally important. Due to the difficulty of recruiting trials in this ultra-rare entity, most trials are powered to show a difference in ORR against historical control data, or even to show any activity. Of note, older trials might use definitions of response other than Response Evaluation in Solid Tumors-RECIST. Lastly, ITMIG has proposed a modified version of RECIST (mRECIST), which leads to similar response rates compared to conventional RECIST and has thus not translated to clinical practice or to most trials [13].

## 3. First-Line Therapy for Thymoma: The Role of Anthracyclines

Platinum-based combination therapy is the accepted standard backbone for *exclusive chemotherapy* (palliative first-line therapy). The underlying studies were extensively reviewed elsewhere [14]. All regimens have been established in single-arm trials. No comparative trials have been conducted. In thymoma, the following options exist: cyclophosphamide + doxorubicin + cisplatin (CAP) +/– prednisone; cyclophosphamide + doxorubicin + cisplatin + vincristine (ADOC); carboplatin + paclitaxel; cisplatin + etoposide +/– ifosfamide. Guidelines prefer anthracycline (AC)-containing regimens based on higher response rates in published prospective and retrospective studies (69% in regimens containing AC, 38% in regimens without) [14]. At the same time, evidence that ACs prolong progression-free (PFS) or overall survival (OS) is lacking. One retrospective study confirmed the difference in ORR but did not find a significant difference in PFS or OS thus challenging this standard (abstract only) [15]. The power to detect differences in OS is limited in any TET analysis due to sample size, but absence of a meaningful PFS improvement would be relevant. Full publication of these results must be awaited, and potential bias must be assessed.

In other solid tumors, small cell lung cancer in particular, AC-containing regimens have also been associated with higher response rates and variable signals with respect to a modest OS benefit compared to platinum-based regimens. Across most trials, hematologic and cardiac toxicity has been higher in the AC arms, including fatal febrile neutropenia, which has led to deprioritization in favor of platinum-doublet therapies [16,17,18,19]. Cardiotoxicity is of particular relevance to patients with TET given the frequent use of mediastinal radiotherapy (RT). Additive or possibly super-additive cardiotoxicity from RT and AC has been well established in diseases like Hodgkin’s lymphoma (25-year risk of heart failure after ≥21 Gy RT +/– AC was 33% vs. 13%) [20] and breast cancer (internal mammary chain RT + AC was associated with a hazard ratio of 9.2 for heart failure compared to 4.2 for AC and 1.6–2.4 for RT alone) [21]. The first major study of cardiovascular outcomes in patients with TET showed comparatively high rates of different cardiovascular diseases (11% at 5 years, 20% at 10 years, 28% at 14 years) and suggested super-additive toxicity of RT and AC (HR for cardiovascular disease 2.9 (RT) vs. 4.6 (AC) vs. 12.2 (RT + AC)) [22], which was most pronounced when RT involved the left ventricle [23]. Moreover, both randomized trials and population data indicate that ACs are associated with increased rates of secondary hematologic malignancies as compared to non-AC based regimens, although the absolute risks remain below 0.5% [18,24,25].

In summary, in the absence of comparative trials, it is likely that AC regimens are associated with more acute (hematologic) and late (cardiac) toxicity compared to non-AC regimens. The impact of late cardiac toxicity in the setting of *exclusive* (palliative) therapy of thymoma remains largely unclear. It is worth noting, however, that median OS for unresectable thymoma varies widely depending on patient selection, ranging from 2–9 years [5,15], but may well be within the range of 5 to 10 years where Nardi Agmon et al. detected 10–20% cardiovascular events [22]. Along this line, the risk for cardiovascular events in their analysis was independent of tumor stage. On the other hand, it is also likely that ACs are associated with higher response rates in thymoma. Whether the risk benefit ratio for adding ACs to definitive systemic therapy in all patients with advanced thymoma is favorable remains uncertain and needs to be further studied.

Established strategies to mitigate AC-associated cardiotoxicity in other entities which could be explored in TET include liposomal formulations of doxorubicin and dexrazoxane [26]. While dexrazoxane is approved for cardioprotection in patients receiving more than 300 mg/m^2^ doxorubicin, guidelines for young cancer patients set lower thresholds in patients who are also exposed to chest radiation [27,28]. Such a strategy could better reflect potential super-additive toxicity in TET [22] and exploit a specific mechanism of cardioprotection of dexrazoxane from radiotherapy that has been proposed based on an animal model [29].

## 4. First-Line Therapy for Thymic Carcinoma: New Combination Regimens

The standard first-line palliative therapy for TC is carboplatin + paclitaxel based on two prospective single-arm trials: WJOG4207L [30] and ECOG E1C99 [31]. Results are summarized in Table 1. In addition, reviews of published prospective and real-world studies do not indicate that AC-based regimens increase ORR or PFS [32]. Real-world studies have confirmed the efficacy of carboplatin + paclitaxel with similar ORR and PFS [8,33,34]. Recently, the addition of either immune checkpoint inhibitors (ICI) or antiangiogenic agents to this standard has been investigated (Table 1).

The addition of atezolizumab to carboplatin and paclitaxel in first-line palliative therapy was investigated in the single-arm MARBLE trial (Path–/Rad+) [35]. The trial was positive regarding the primary endpoint of objective response rate, which was 56% in 48 patients and significantly higher than the predefined historical control threshold of 30%. The authors state that the trial was not designed to show superiority over carboplatin and paclitaxel alone. A similarly designed trial using the PD-L1 inhibitor adebrelimab with carboplatin and nab-paclitaxel successfully passed the first stage of a two-stage design (ORR 66.7%) and continues enrollment (Path/Rad not reported) [36]. Both trials reported a PFS of about 10 months which is numerically higher than 5–8 months historically reported with carboplatin + paclitaxel. Overall survival is not yet mature and the proportion of patients achieving long-term disease control is not yet clear. The incidence of immune-related adverse events (irAE) of grade 3 and higher were 17–18% in both trials. As a reference, approved immunochemotherapy in non-small cell lung cancer is associated with grade 3 or higher irAE in 2.3% (cemiplimab [37]), 8.9% (pembrolizumab [38]), and 8.5% (atezolizumab [39]) of cases, respectively.

In the same setting, the single-arm, phase 2 RELEVENT trial (Path+/Rad+) investigated the addition of ramucirumab to carboplatin and paclitaxel. The study was positive for the primary endpoint of centrally reviewed ORR reaching 57.6% (95% CI 39.2% to 74.5%, *p* < 0.0001), which was shown to be significantly improved over the set historical threshold of 20%. Median PFS (18.1 months, 95% CI 10.8 to 52.3) and OS (43.8 months, 95% CI 31.9 to NR) compared very favorably to historic references and other current trials (Table 1). Importantly, restrictions of cross-trial comparison including testing multiplicity and selection bias do not allow drawing valid external conclusions from this comparison. Patients numbering 11% experienced serious adverse events related to ramucirumab. Moreover, the addition of ramucirumab was investigated in the randomized, open-label phase 2 trial SWOG S1701 against carboplatin + paclitaxel (Path–/Rad–) [40]. The trial was stopped prematurely and was thus not sufficiently powered to assess the prespecified hypothesis of improving median PFS by a hazard ratio (HR) of 0.5. The 20 enrolled patients showed longer PFS (hazard ratio = 0.51, 80% CI: 0.24–1.09, *p* = 0.13) and higher ORR (88% (seven of eight, 80% CI: 59–99%) vs. 40% (four of ten, 80% CI: 19–65%)) in the intervention arm. The differences were not statistically significant and must be interpreted with caution due to the small sample size. Taken together with the RELEVENT trial, they support that adding ramucirumab to chemotherapy increases efficacy.

**Table 1:** First-line therapy options for patients with thymic carcinoma. *: abstract only. CI: confidence interval. mOS: median overall survival. mPFS: median progression-free survival. NA: not available. NR: not reached. ORR: objective response rate. Path: pathological review. q3w: every 3 weeks. Rad: radiological review. RECIST: Response evaluation in solid tumors. TC: thymic carcinoma. THY: thymoma. TRAE: treatment-related adverse event.

Regimen	Name, Year, Reference	Entity	Central Review	No. of Patients	Primary Hypo-thesis	Primary Outcome	ORR	Response Definition	mPFS (Months)	mOS (Months)	Discontinuation Due to TRAE
**Carboplatin AUC 6 + paclitaxel 200 mg/m^2^, q3w, 6x**	WJOG4207L, 2015, [30]	TC	Path+/ Rad+	39	ORR > 20%	Positive trial. ORR 36% (95% CI 21–53%)	36% (95% CI 21% to 53%)	RECIST 1.1	7.5 (95% CI, 6.2 to 12.3)	NR	8%
**Carboplatin AUC 6 + paclitaxel 225 mg/m^2^,** **q3w, x6**	E1C99, 2010, [31]	THY/TC	Path+/ Rad–	THY: 21 TC: 23	THY: ORR 60% vs. 40% TC: ORR 45% vs. 20%	THY: 42.9% (90% CI, 24.5% to 62.8%) TC: 21.7% (90% CI, 9.0% to 40.4%)	ORR 22% (90% CI 9% to 40%)	RECIST 1.0	THY: 16.7 (95% CI, 7.2 to 19.8) TC: 5 (95% CI 3 to 8)	THY: NR TC: 20.0 (95% CI, 5.0 to 43.6)	NA
**Atezolizumab 1200 mg + carboplatin AUC 6 + paclitaxel 200 mg/m^2^,** **q3w, x6 +** **atezolizumab maintenance**	MARBLE, 2025, [35]	TC	Path–/Rad+	48	ORR > 30%	Positive trial. ORR 56% (95% CI 41–71; *p* < 0.0001)	56% (95% CI 41% to 71%)	RECIST 1.1	9.6 (95% CI 7.7 to 14.8)	NR	13%
**Adrebelimab 20 mg/kg + carboplatin AUC 5 +** **nab-paclitaxel 260 mg/m^2^, q3w, x4** **–** **6 +** **adreblimab maintenance**	Interim analysis 2025 *; still ongoing, [36]	TC	NA	18	ORR	NR	66.7% (12/18)	NA	10.2 (95% CI, 8.8 to 11.6)	NR	11%
**Ramucirumab 10 mg/kg + carboplatin AUC 5 + paclitaxel 200 mg/m^2^,** **q3w, x6 +** **ramucirumab maintenance**	RELEVENT, 2024, [2]	TC	Path+/ Rad+	35	ORR > 20%	Positive trial. *p* < 0.0001	57.6% (95% CI 39.2% to 74.5%)	RECIST 1.1	18.1 (95% CI 10.8 to 52.3)	43.8 (95% CI 31.9 to NR)	17%
**Carboplatin AUC 6 + paclitaxel 200 mg/m^2^, q3w, x6** **+/– ramucirumab 10 mg/kg, q3w incl. maintenance**	SWOG S1701, 2024 (terminated early, slow accrual), [40]	TC	Path–/Rad–	20	PFS HR 0.5	Not evaluable/not positive. mPFS HR = 0.51 (80% CI: 0.24 to 1.09, *p* = 0.13)	Ramucirumab: 88% (7/8, 80% CI: 59% to 99%) vs. Control: 40% (4/10, 80% CI: 19% to 65%)	RECIST	8 (80% CI: 7–11) (Ramucirumab) vs. 7 (control) (80% CI: 6–8)	NR	2/8 (25%) vs. 2/9 (22%)

## 5. Treatment Options for Recurrence After Platinum-Based Definitive Systemic Therapy (2nd Line)

The only approved therapy for TET after platinum failure is lenvatinib, which has a label for TC in Japan. Several other drugs are active in TET including chemotherapy, anti-PD-1/PD-L1 antibodies, antiangiogenic drugs, the CDK4/6 inhibitor palbociclib, and the mTOR inhibitor everolimus. Response rates range between about 15–40% and median PFS between 4 and 16 months. The evidence is summarized in Table 2. In addition, the ESMO guideline also recommends considering re-exposure to platinum-based combination therapy. Although evidence is limited [41,42,43] given the analogy to other entities, it seems to be a valid treatment option in patients with response to first-line platinum treatment and a sufficiently long treatment-free interval. When re-exposing to anthracyclines, the maximum cumulative dose and the use of dexrazoxane must be considered. In addition to limited efficacy, several drugs induce considerable toxicity as indicated by 17–26% of patients discontinuing treatment with drugs like everolimus, multi-kinase inhibitors, or immunotherapy due to adverse events (Table 2). Beyond discontinuation rates, tolerability is difficult to compare across trials as the reporting and management of adverse events vary. The subjective burden of adverse events and quality of life is not available at all. Given all the limitations in cross-trial comparison in general and for TET in particular as discussed above, no clear standard 2nd line treatment has emerged.

Octreotide + prednisone for treatment of TET expressing somatostatin receptor (SSR) remains part of several guidelines. Expression of SSR is seen in THY, rarely in TC. Octreotide monotherapy has a low early response rate of 11% [44]. Its contribution to delaying progression is unknown since it has only been studied in combination with corticosteroids [45]. Steroids are known to be active as monotherapy in THY with response rates of about 50% and higher but to be associated with considerable toxicity at the usual doses of 0.6 mg/kg prednisone QD or pulsed therapy with 1 g methylprednisolone [46,47,48,49]. Both the actual benefit from octreotide and the optimal dose for steroids need to be better characterized.

The results of several trials with anti-PD-1/PD-L1 antibody monotherapy (immune checkpoint inhibitor—ICI) have been extensively reviewed elsewhere [50]. In short, ICI monotherapy is associated with a low but consistent response rate of about 18% and a median progression-free survival of about 4–6 months. An outlier of 11.7 months PFS, observed with atezolizumab in THY, may be attributed to chance in the small sample set of 13 individuals (95% CI 3.22–37.22) [51]. Importantly, due to high rates of ≥grade 3 immune-related adverse events (irAE), ICIs are generally not recommended in patients with THY (58.3%) and may be used with caution in patients with TC (17.1%). The long-term follow-up (median 4.9 years) of one of the pembrolizumab trials indicates that ICIs might lead to long-term disease control in some patients [52]. All nine responding patients (total *n* = 40) developed progressive disease after a median duration of response of 2.99 years. Two patients continued pembrolizumab beyond progression, and one received local ablative radiotherapy for oligo-progression and continued with pembrolizumab. Responders had longer survival compared to non-responders. Four patients discontinued treatment for reasons other than progressive disease, later progressed, and received re-challenge pembrolizumab. Two of these patients responded again.

The recently published single-arm PECATI trial introduced lenvatinib (20 mg QD) combined with pembrolizumab as a new treatment option for recurrence after platinum-based chemotherapy [53]. The trial included patients with TC and B3 thymoma following platinum failure who were not previously exposed to antiangiogenic therapy. The trial was positive for the primary endpoint, demonstrating a PFS rate at 5 months of 88.4% (90% CI 79.8–96.7) and ruled-out the null hypothesis of 50% or lower which was based on historic pembrolizumab monotherapy data (mPFS 4–6 months). It may be criticized, that the higher historic PFS of lenvatinib monotherapy (24 mg QD) might rather have been used as a stronger historic comparator (REMORA [54]: 9.3 months (95% CI 7.7–13.9); real-world [55]: 10.2 months (95% CI 7.0–13.2)). Nevertheless, although the response rate remained low in PECATI (23.3%), the median PFS of 14.9 months (95% CI 10.6-NE) compares favorably also to lenvatinib monotherapy and other published regimens. Toxicities did not seem to be super-additive compared to monotherapy; 26% discontinued treatment due to treatment-related adverse events (TRAE).

**Table 2:** Second and later-line therapy options for patients with thymoma and thymic carcinoma. All data are from single-arm prospective trials, except for data marked with # indicating retrospective analysis. +: abstract only. *: After 6 weeks of octreotide monotherapy, prednisone was added only in patients with stable disease. BID: twice daily. CI: confidence interval. D1 + D8: Day one and day eight of each cycle. LAR: long-acting release. mOS: median overall survival. mPFS: median progression-free survival. NA: not available. NR: not reached. ORR: objective response rate. Path: pathological review. q3w: every 3 weeks. QD: quotidian. Rad: radiological review. RECIST: Response evaluation in solid tumors. TC: thymic carcinoma. THY: thymoma. TID: thrice daily. TRAE: treatment-related adverse event.

Regimen	Name, Year, Reference	Entity	Central Review	No. of Patients	Hypothesis	Primary Outcome	ORR	ORR Definition	mPFS (Months)	mOS (Months)	Discontinuation Due to TRAE
**Everolimus 10 mg QD**	2017, [56]	Thy/TC	Path–/Rad–	50	DCR 60% vs. 40%	Positive trial (DCR 88%)	12%	RECIST 1.1	Total: 10.1 Thy: 16.6 TC: 5.5	Total: 25.7 Thy: NR TC: 14.7	18%
**Gemcitabine 1000 mg/m^2^ D1 + D8 + Capecitabine 650 mg/m^2^ BID D1–14, q3w**	2014, [57,58]	Thy/TC	Path+/Rad–	30	ORR ≥ 35% vs. <15%	Positive trial	40%	RECIST	Total: 11 Thy: 11 TC: 6	NR	NA
Octreotide 0.5 mg TID (+ prednisone 0.6 mg/kg QD) *	2004, [44]	Thy/TC	Path–/Rad–	38	ORR ≥ 30%	Positive trial.	Total: 31.6% (95% CI: 17.5% to 48.7%) Octreotide alone: 10.5% TC: 0%	PR: ≥ 50% reduction PD: ≥25% increase	Total: 9.2 Octreotide monotherapy: 2	NR	NA
**Octreotide LAR 30 mg q2w + prednisone 0.6 mg/kg QD**	2016, [59]	Thy/TC	Path–/Rad–	17	NA	NA	88%	PR: ≥ 20% reduction PD: ≥25% increase	NA	NA	18%
**Pemetrexed 500 mg/m^2^ q3w, maximum 6 cycles**	2018, [60]	Thy/TC	Path–/Rad–	27	ORR > 20% vs. <5%.	Positive trial	Total: 19.2% (95% CI: 6.3 to 38.1) Thy: 4/15 (27%) TC: 1/11 (9%)	RECIST	10.6 (95% CI: 2.9 to 13.4)	28.7 (95% CI: 9.8 to 47.2)	NA
**S1; 80,100 or 120 mg BID D1–14, q3w**	# 2019, [61]	TC	Path–/Rad-	44	NA	NA	30%	mRECIST (ITMIG)	6 (95% CI: 7.0 to 13.9)	15 (95% CI: 13.2 to 21.6)	5%
**Lenvatinib 24 mg QD**	REMORA, 2020, [54]	TC	Path–/Rad+	42	ORR ≥ 25%	Positive trial.	38% (90% CI: 25.6 to 52.0)	RECIST 1.1	9.3 (95% CI: 7.7 to 13.9)	NR	17%
**Pembrolizumab 200 mg q3w**	2019, [62]	Thy/TC	Path–/Rad-	33	ORR ≥ 25% vs. 5%	NA.	21.2% (95% CI: 10.7 to 37.8)	RECIST 1.1	6.1 (95% CI: 5.3 to 6–9)	NA	24%
**Pembrolizumab 200 mg q3w**	2018, [63]	TC	Path+/Rad+	40	ORR ≤ 5% vs. ≥20%	Positive trial.	22.5% (95% CI 10.8–38.5	RECIST 1.1	4.2 (95% CI: 2.9 to 10.3)	24.9 (95% CI:15.5 to NR)	NA
**Nivolumab 240 mg q2w**	NIVO-THYM, 2023, [64]	Thy/TC	Path–/Rad+	49	PFS rate at 6 months 60% vs. 40%	Negative trial. PFS rate at 6 months 35% (95% CI 22% to 50%)	14%	RECIST 1.1	6.2 (95% CI: 3.1 to 10.4)	21.3 (95% CI: 11.6 to NR)	20%
**Ipilimumab 1 mg/kg q6w + Nivolumab 240 mg q2w**	NIVO-THYM +, 2025, [65]	Thy/TC	Path–/Rad+	56	PFS rate at 6 months	Negative Trial. 21.6% PFS rate at 6 months	17.7%	RECIST 1.1	3.2 (95% CI: 2.1 to 3.6)	22.0 (95% CI: 16.6 to NR)	22%
**Sunitinib 50 mg QD D1–28, q6w**	2015, [66]	Thy/TC	Path–/Rad-	41	ORR ≥ 25% in TC ORR ≥ 30% in Thy	TC: positive Thy: negative	TC: 26% (90% CI: 12.1 to 45.3) Thy: 6% (95% CI: 0.2 to 30.2)	RECIST 1.1	TC: 7.2 (95% CI: 3.4 to 15.2) Thy: 8.5 (95% CI: 2.8 to 11.3)	TC: NR Thy: 15.5 (95% CI 12.6 to NR)	TC: 21% Thy: 13%
**Sunitinib 50 mg QD D1** **–** **28, q6w**	STYLE, 2023, [67]	Thy/TC	Path–/Rad–	44	ORR ≤ 5% vs.- ≥25%	Thy: negative. TC: positive	THY: 0% (90% CI: 0.0% to 22.1%) TC: 21.7% (90% CI: 9.0% to 40.4%)	RECIST 1.1	Thy: 7.7 (95% CI: 2.4 to 45.5) TC: 8.8 (95% CI: 5.3 to 11.1)	Thy: 47.9 (95% CI: 4.5 to NR) TC: 27.8 (95% CI: 13.2 to 53.2)	Thy 18% TC 8%
**Atezolizumab 1200 mg, q3w**	2022, [51]	Thy/TC	Path–/Rad–	25	Non-progression at 18 weeks (NPR-18) ≥ 40%	Thy: positive. NPR-18: 76.9% (95% CI: 46.2 to 95.0) TC: Not evaluable. NPR-18: 41.7 (95% CI: 15.2 to 72.3)	Thy 23.1 (95% CI: 5.0 to 53.8) TC: NA	RECIST 1.1	Thy 11.76 (95% CI: 3.22 to 37.22) TC: NA	NE	NA
**Avelumab 10 mg/kg q2w + axitinib 5 mg BID**	2022, CAVEATT, [68]	Thy/TC	Path–/Rad+	33	ORR ≤ 20% vs.- ≥40%	34% (90% CI 21–50)		RECIST 1.1	7.5 (90% CI: 3.7 to 10.0)	26.6 (90% CI 17.0 to 30.0	12%
**Lenvatinib 20 mg QD + pembrolizumab 200 mg q3w**	2025, PECATI, [53]	Thy/TC	Path–/Rad–	43	PFS rate at 5 months ≥ 68.6% vs. ≤50%	Positive trial. PFS rate at 5 months: 88.4% (90% CI 79.8 to 96.7)	23.3% (95% CI:11.8 to 38.6)	RECIST 1.1	14.9 (95% CI: 10.6– to NR)	NR	26%
**Palbociclib 125 mg QD, D1** **–** **21, q4w**	2023, [69]	Thy/TC	Path–/Rad–	48	PFS rate at 6 months 50% vs. 30%	Positive trial. PFS rate at 6 months: 60.2%	12.5%	RECIST 1.1	11.0 (95% CI: 4.6 to 17.4)	26.4 (95% CI: 9.4 to 43.4)	0%
**Amrubicin, 35–40 mg/m^2^ D1-3, q3w**	2019, [70]	Thy/TC	Path-/Rad-	33	ORR ≤ 5% vs. ≥20%	Positive trial. ORR 18% (90% CI: 8.2% to 32.8%)	Total: 18% (90% CI: 8.2% to 32.8%) THY: 29% TC: 11%	RECIST 1.1	7.7 (90% CI: 6.2 to 12.7)	29.7 (90% CI: 18.0 to 54.1)	15%
**Anlotinib 10–12 mg QD D1** **–** **14, q3w**	# 2023, [71]	Thy/TC	Path-/Rad-	50	NA	NA	THY: 33% (11/33) TC: 41% (7/17)	RECIST 1.1	THY: 7 (95% CI: 5.9 to 10.2) TC: 6 (95% CI: 4.6 to 9.3)	NA	16%

## 6. Potential Treatment Sequences in Thymoma

In thymoma, first-line therapy is platinum-based and potentially involves anthracyclines as discussed above. Treatment options beyond platinum including potential re-exposure are everolimus, palbociclib, anlotinib, and chemotherapy (gemcitabine + capecitabine, pemetrexed, amrubicin). The latter is associated with numerically higher response rates (Table 2). Numerically, everolimus was associated with the longest median PFS of 16.6 months (95% CI, 9.8 to 29.8 months) in a prospective phase 2 trial [56]. Octreotide + prednisone may be used in tumors expressing somatostatin receptor but is less preferable given the limitations described above. Steroids without octreotide may be considered to induce response in symptomatic patients given the response rates described above. Currently, no inference can be made to the optimal sequencing. Selection may be guided by patients’ individual risk to be affected by specific toxicities, access, and his or her preference.

## 7. Potential Treatment Sequences in Thymic Carcinoma

In thymic carcinoma, determining the optimal sequence has become a challenge since both antiangiogenic drugs and ICIs have been introduced to first-line combination therapy (ramucirumab or atezolizumab + platinum). In later lines, new combinations could now potentially be used (pembrolizumab + lenvatinib; axitinib + avelumab) in addition to monotherapy options (lenvatinib, sunitinib, ICI), and chemotherapy (platinum re-exposure; gemcitabine + capecitabine, S1, pemetrexed, amrubicin). The optimal sequence is unknown. In other entities, especially lung cancer, treatment paradigms have shifted towards prioritizing the most effective therapies and combinations early over sequential monotherapies due to substantial attrition rates between treatment lines [72,73,74,75]. While this logic cannot be transferred to the indolent course of thymoma (5-year OS 58.5% for stage IV [5]), it might apply to TC which has a prognosis (5-year OS of inoperable TC 24% [7]) comparable to that of lung cancer (5-year OS of EGFR+ NSCLC 28% [76]). In a corresponding French real-world analysis, 66% of patients with TC who started exclusive, i.e., palliative first-line, chemotherapy went on to receive second line systemic therapy after a median of 27 months of follow-up [8]. Here, we provide arguments for using different sequences, all of which are hypothesis-generating.

### 7.1. Thymic Carcinoma, First-Line Scenario A: Ramucirumab + Platinum Doublet (RELEVENT)

*Pro:* This regimen was associated with a numerically higher PFS (18.1 months, 95% CI 10.8–52.3) and similar ORR compared to atezolizumab + platinum doublet (9.6 months, 95% CI 7.7–14.8) or any other regimen. The median OS of 43.8 months (95% CI 31.9 months-not reached) compares favorably to real-world data of stage IV TC (33 months [7] and 30.7 months [32]). It was associated with less TRAE of grade 3 to 5 (11.6%) compared to MARBLE (75%; including 15% irAE).

*Contra*: If resectability is induced, surgical complications might be increased in the context of VEGF inhibition. Validity of comparing OS and safety data from a selected trial population to real-world data and other trials is limited. It is possible that standard 2nd line therapies targeting VEGF, including lenvatinib and sunitinib, might be less effective following ramucirumab (see below).

*Implications for sequencing*: It is unknown whether antiangiogenic second-line therapy following ramucirumab pretreatment is effective. Lenvatinib was the most commonly used 2nd line therapy in RELEVENT, but PFS2 was not reported. Activity of the anti-VEGF antibody bevacizumab is not lost across lines in colorectal carcinoma [77,78]. However, in a post hoc analysis of CAVEATT (axitinib + avelumab in previously treated THY/TC), efficacy was low in patients with previous antiangiogenic therapy (ORR 15% (3–41) vs. 47% (90% CI 27–68)). Therefore, patients with previous antiangiogenic therapy were excluded from PECATI (lenvatinib + pembrolizumab). This may be an argument for prioritizing other options like pembrolizumab, palbociclib, or chemotherapy in this setting.

### 7.2. Thymic Carcinoma, First-Line Scenario B: Atezolizumab + Platinum Doublet (MARBLE)

*Pro:* ICIs probably have the potential to induce long-term disease control in TC [7] which cannot be expected from other treatment options. Early use of ICIs maximizes the chance of every patient developing long-term disease control given the risk of not reaching 2nd line therapy. While toxicity is an issue, it is manageable as indicated by the similar rates of patients discontinuing due to toxicity in MARBLE (13%) and RELEVENT (17%).

*Con:* Overall survival has not been reported in MARBLE. The PFS curve does not show a clear plateau, so the real potential for long-term disease control of this regimen remains unknown. Given the longer PFS when used in combination with chemotherapy in first line as opposed to later monotherapy, the exposure of patients not sensitive to ICIs and the associated risk for irAEs is increased as compared to later line ICI monotherapy, where therapy can be quickly discontinued if it is not effective.

*Implications for sequencing:* PECATI and CAVEATT did exclude patients with prior ICI treatment. Re-exposure to ICIs including combination with antiangiogenic agents has shown low efficacy across several trials in lung cancer [79]. Switching drug class to antiangiogenic therapy, palbociclib, or chemotherapy may thus be preferable.

### 7.3. Thymic Carcinoma, First-Line Scenario C: Carboplatin + Paclitaxel

*Pro:* No valid evidence exists showing that either combination regimen prolongs OS, arguably the most relevant endpoint in non-resectable TC. Both physical and financial toxicity are minimized.

*Con*: This strategy risks losing patients between lines and depriving them of exposure to active substance classes, thus potentially compromising overall prognosis. Hence, it may not be adequate for most patients with TC who have a clinically aggressive course of disease. Arguing strictly with the formal lack of a valid OS benefit, as often done by insurances and regulators, does not adequately reflect the difficulty of obtaining valid data in this ultra-rare population and is thus not in the patients’ best interest.

*Implications for sequencing*: If efficacy is to be prioritized, lenvatinib + pembrolizumab could be selected based on the numerically longest PFS. In absence of a head-to-head comparison regarding efficacy, one could also choose the regimen with the best tolerability. All regimens have significant but manageable toxicities (see Table 2 for discontinuation rates). At the lowest end of the spectrum is palbociclib with 0% discontinuation rate due to toxicity [69]. The percentage of patients experiencing grade III or higher toxicities was 37% in PECATI, 64% in a lenvatinib real-world study [55], and 15% for pembrolizumab [62]. Safety data for the specific regimen of gemcitabine and capecitabine used in TC is limited [57]. How this data translate to patient experience is unclear; i.e., grade 2 fatigue or hand-foot syndrome is likely perceived to be more severe than grade 3 hypertension or neutropenia. Quality-of-life data are not available.

## 8. Personalized Therapy and Predictive Biomarkers

The molecular landscape and the resulting low potential of targeted therapy in TET has been reviewed recently [80]. Due to the rarity of TET, patients are often eligible to enroll in personalized oncology programs involving extensive genomic assessment. Results thus far have been discouraging: One study reported 8 of 65 enrolled cases with TET benefiting from the recommended therapy, which was sunitinib in five and pembrolizumab in two cases, being standard therapies anyway [81].

Mutations in KIT and PDGFRA have been described in 7–20% and 0–6% of TC, respectively, but not in THY [81,82,83,84,85,86]. Response of TC with KIT mutations to KIT inhibitors like imatinib, sorafenib, and sunitinib has been described in case reports [85,87,88]. Publication bias has to be observed when setting expectations for response in a patient with TET and KIT mutation. In case of progression to an early generation KIT inhibitor in a patient with KIT-mutant TC, resistance testing and switch to a next-generation KIT inhibitor in analogy to gastrointestinal stroma tumors may be considered.

Expression of HER2 has been described in 5% to 58% of TC [89]. In most studies, the intensity as reported in breast cancer has not been described. Given the approval of the anti-HER2 antibody–drug conjugate trastuzumab deruxtecan (T-DXd) for previously treated solid tumors with HER2 overexpression (IHC 3+), it seems advisable to test TC patients in clinical practice until further data are available. In the underlying trial, however, no patient with TC was included [90].

Mutations in HRAS and NRAS have been found in individual patients with TET [81,86]. As panRAS inhibitors move to the clinic, this might become relevant for some patients in the near future [91].

## 9. Limitations

This review is based on a non-systematic search and is biased by the selection of the authors. All comparisons and prioritizations regarding different drug regimens discussed above are based on cross-trial comparisons of small single-arm trials and retrospective series that often do not report mature survival data. They are therefore prone to selection and publication bias, confounding, and to random effects, and must be interpreted cautiously. In addition, they do not account for statistical errors and testing multiplicity. Importantly, trials differ in whether they enrolled patients with TET in general or specific subtypes only and whether a central pathology and radiology review was conducted. Finally, different primary endpoints have been chosen across trials, and most were not designed to show superiority even over a historic control, but rather just any activity. We aimed to provide transparency over these aspects in Table 1 and Table 2.

## 10. Conclusions

Beyond platinum-based chemotherapy, an increasing number of drugs have shown activity in TET, most importantly antiangiogenic agents and immune checkpoint inhibitors. The major academic and clinical needs are, first, to find a definition of clinically and statistically meaningful improvement over a given standard that is acceptable to physicians, insurances, and regulators and that is feasible to obtain in ultra-rare TET. In many countries, the new therapies reviewed here will be difficult to transfer to clinical practice due to regulatory constraints. For the scientific community, it is difficult to confidently define current standards given the nature and design of recent trials. Bayesian trial designs, allowing for randomization also in small sample sets, may be an option to explore [92,93]. Existing real-world data, e.g., from ITMIG, should be used to benchmark new treatment strategies and to uncover potential biases when comparing data sets from different regions. Second, in a situation with several drugs of comparable efficacy, tolerability should become a criterion for selection. Thus, tolerability and quality of life need to be reflected as key endpoints in the design of future trials. The use of anthracyclines in THY requires re-evaluation considering both added efficacy and existing strategies for cardioprotection. Third, of course, new therapeutic strategies are needed to improve survival. Patients should be treated in clinical trials, whenever possible. Oncologists are encouraged to associate with national and international networks for rare tumors and TET to exchange experience, drive clinical research, and for political lobbying [94].

## 11. Future Directions

It has now been well established that both antiangiogenic agents and anti-PD-1/anti-PD-L1 antibodies are active in TET. Several trials aim at further optimizing the use of these two strategies, especially in combination. The single-arm ARTEMIS trial [95] is currently enrolling previously untreated patients with advanced TC for a treatment of four cycles induction therapy with carboplatin AUC 5, paclitaxel 175 mg/m^2^, pembrolizumab 200 mg q3w, and lenvatinib 8 mg QD, followed by maintenance pembrolizumab and lenvatinib 20 mg. The primary endpoint is ORR, which is aimed to be improved over a historical threshold of 35%. The combination of anlotinib and the anti-PD-1 antibody tislelizumab is being investigated in a phase 2 trial in advanced TET (NCT06838910). Ivonescimab is a tetravalent Fc-silent bispecific anti-VEGF/anti-PD-1 antibody with a new cooperative mode of target engagement [96] (abstract only). It has shown superiority over conventional anti-PD-1 antibodies in selected populations in NSCLC [97]. Trials in TC are ongoing, both as monotherapy (NCT06980077) and in combination with chemotherapy (NCT06750952). Moreover, new drugs like the antiangiogenic agent and multi-kinase inhibitor KC1036 (NCT05683886, [98,99], 86: abstract only) and the small-molecule pyrophosphate–platinum conjugate PT-112, an inducer of immunogenic cell death [100,101,102] (89–90: abstract only), are being explored.

To advance systemic therapy of TET, it is essential to deepen our understanding about underlying biological mechanisms and to explore upcoming biomarkers from other tumors. TROP2 has been shown to be expressed in thymic carcinoma [103]; hence, several studies have been initiated involving anti-TROP2 antibody–drug conjugates including sacituzumab govitecan (NCT06248515) and sacituzumab tirumotecan (NCT07324629, NCT07343453). Similarly, mesothelin is expressed in TC [104,105] and is targeted using CAR NK (NCT07598955) and CAR T cells (NCT06885697, [106]). With respect to neuroendocrine TC, anti-DLL3 T-cell engagers like tarlatamab or obrixtamig are a promising new therapy. Although no data are available from patients with TET, obrixtamig has shown activity in a number of extrapulmonary NEC, especially when DLL3 was expressed in more than 50% of tumor cells [107,108] (96: abstract only). In addition, it has recently been described that many TET, TC in particular, show high expression of the immunoproteasome, in contrast to squamous cell carcinoma of other origin. This renders them susceptible to therapy with proteasome inhibitors both in vitro and in vivo. Clinical validation is ongoing [109].

## Data Availability

No new data were created or analyzed in this study. Data sharing is not applicable to this article.
